# Binarization Parameters Affect Quantitative Choriocapillaris Biomarkers Derived from Optical Coherence Tomography Angiography

**DOI:** 10.3390/biomedicines14102395

**Published:** 2026-10-20

**Authors:** Anait S. Khalatyan, Yusef Yusef, Konstantin S. Avetisov, Sergey A. Smolin, Vladimir I. Siplivy, Anna E. Aslamazova, Ludmila V. Reznikova, Liubov V. Machekhina, Alexandra A. Melnitskaia, Irina D. Strazhesko

**Affiliations:** 1 Krasnov Research Institute of Eye Diseases, Moscow 119021, Russia; info@eyeacademy.ru (Y.Y.); avetisov.k.s@gmail.com (K.S.A.); smolin_s_a@staff.sechenov.ru (S.A.S.); 2 The Department of Ophthalmology, Sechenov First Moscow State Medical University ( Sechenov University), Moscow 119048, Russia; siplivyy_v_i@staff.sechenov.ru (V.I.S.); aslamazova_a_e@staff.sechenov.ru (A.E.A.); reznikova_l_v@staff.sechenov.ru (L.V.R.); 3 Russian Gerontology Research and Clinical Centre, Pirogov National Research Medical University, Moscow 129226, Russia; machehina_lv@rgnkc.ru (L.V.M.); melnickaya_aa@rgnkc.ru (A.A.M.); strazhesko_id@rgnkc.ru (I.D.S.)

**Keywords:** aging, choriocapillaris, optical coherence tomography angiography, Phansalkar thresholding, binarization, flow deficits, image processing

## Abstract

**Background/Objectives:** Quantitative choriocapillaris biomarkers derived from optical coherence tomography angiography (OCTA) depend on binarization settings. We assessed Phansalkar parameters using chronological age as an exploratory physiological reference. **Methods:** Seventy participants (70 eyes) in three age groups (22–40, 41–60, and 61–83 years) underwent swept-source OCTA. In Fiji, the window radius (R = 2–6 pixels) was varied at k = 0.25 and r = 0.50; 24 k–r combinations were subsequently examined at R = 3. Age-group effects were evaluated using ANOVA, η^2^, false discovery rate (FDR) correction, and permutation max-statistic testing. **Results:** Across radii, only large flow-deficit counts (FDN5000 and FDN10000) consistently differed between age groups, peaking in middle age. Among the five radii, η^2^ was largest at R = 4 for FDN5000 (0.164) and at R = 3 for FDN10000 (0.261). The common working set (R = 3, k = 0.25, r = 0.50) maximized the mean η^2^ of both counts. At this setting, FDN10000 remained significant after family-wise correction (*p* = 0.001), whereas FDN5000 did not (*p* = 0.060). **Conclusions:** Parameter choice affects choriocapillaris quantification. The identified setting is an exploratory, protocol-specific candidate and requires external validation.

## 1. Introduction

The choriocapillaris is the innermost layer of the choroid, located between Bruch’s membrane and the medium and large choroidal vessels. It comprises anastomosing fenestrated capillaries approximately 20 µm in diameter in the macular region [1,2]. Together with Bruch’s membrane, the choriocapillaris supplies the retinal pigment epithelium and photoreceptors with nutrients and clears metabolic waste from the outer retina into the systemic circulation [3,4].

With aging, the choroid undergoes involutional changes: Bruch’s membrane thickens, and the number of functional choriocapillaris segments decreases [5]. Age-related choroidal diseases are characterized by choroidal remodeling, including choriocapillaris atrophy (“ghost vessels”) [6,7]. These changes contribute to degenerative processes in the macula and to a decline in central vision and quality of life.

Optical coherence tomography angiography (OCTA) is the principal noninvasive imaging modality for the choriocapillaris. It visualizes the motion of blood cells within vessel lumina and provides layer-by-layer en face images. In recent years, a panel of quantitative OCTA biomarkers of the choriocapillaris has been studied across a wide range of diseases [8,9,10].

The first group of biomarkers describes the architecture of the vascular network and includes vessel density and total vascular area, with less frequent use of skeletonized morphometric parameters [11,12,13]. Vessel density (VD, %) is the ratio of perfused capillary area (white pixels of the binary mask) to the total area of the region of interest, expressed as a percentage; reported values range from 44% to 76% depending on the methodology used [14,15,16,17,18]. Vessel area (mm^2^) is the absolute area of perfused capillaries within the region of interest and is mathematically derived from VD [17].

The second group comprises biomarkers of reduced perfusion: the fraction of the layer occupied by regions of reduced flow (flow deficit density, FDD), the mean area of an individual flow deficit (mean flow deficit size, MFDS), and the count of flow deficits (flow deficit number, FDN) [19,20]. This set of indices captures not only the overall extent of perfusion impairment but also the size of hypoperfusion foci. The term “flow deficits” is currently preferred over the historical term “flow voids” because it accounts for regions of substantially reduced but not absent flow and emphasizes that the registered defects should exceed the normal intercapillary spacing of approximately 5 to 20 µm [21,22].

Computation of quantitative biomarkers requires binarization of the choriocapillaris angiograms—that is, conversion of the grayscale image to a black-and-white image. A global threshold may be sensitive to spatial variations in image intensity, whereas local adaptive methods account for neighborhood-level image statistics. Among local methods, the Phansalkar algorithm—a modification of Sauvola’s method that uses local neighborhood statistics—is the most widely used. The Phansalkar threshold is computed as T = mean × (1 + *p* × exp(−q × mean) + k × ((stdev/r) − 1)), where “mean” and “stdev” are the local mean and standard deviation within a window of given radius, “*p*” and “q” are fixed constants (default 2 and 10), and “k” (weight of the standard deviation) and “r” (dynamic range) are tunable parameters referred to in ImageJ as parameter 1 and parameter 2 [23,24].

The values of k and r are frequently not reported, and the window radius is set in a wide range of 1 to 15 pixels across studies [25,26], which precludes direct comparison of published results. The default values of k = 0.25 and r = 0.50, accepted as standard, have no physiological justification and were proposed by Phansalkar et al. for cytological microscopic images of stained cells [23]. Previous methodological studies varied mainly the window radius while keeping k and r at their default values [25,26]; to our knowledge, the joint influence of k and r on choriocapillaris flow-deficit metrics has not been systematically examined.

Prior histological and OCTA studies consistently show that choriocapillaris perfusion deficits increase with age [5,17,19]. Because the binarization parameters R, k, and r influence the derived flow-deficit metrics, chronological age was used as an internal physiological reference to explore parameter-dependent age-group associations, rather than as a reference standard for measurement accuracy or repeatability.

The purpose of the present study was therefore to evaluate how the choice of parameters (R, k, and r) in Phansalkar local adaptive thresholding affects quantitative choriocapillaris OCTA biomarkers, using chronological age as an internal physiological reference for exploratory comparisons of age-group associations across parameter configurations.

## 2. Materials and Methods

### 2.1. Study Design and Ethics

Participants were recruited at the Russian Gerontology Research and Clinical Centre, Pirogov National Research Medical University, and ophthalmic examinations and OCTA imaging were performed at the Krasnov Research Institute of Eye Diseases, Moscow, Russia, between 2022 and 2024. This prospective observational pilot study with retrospective image analysis was reported according to the Strengthening the Reporting of Observational Studies in Epidemiology (STROBE) reporting guideline. The study protocol was approved by the Local Ethics Committee (protocol No. 59 of 13 September 2022) and the study adhered to the tenets of the Declaration of Helsinki. Written informed consent was obtained from all participants after the nature and possible consequences of the study had been explained.

### 2.2. Participants and Eligibility Criteria

Seventy participants (70 eyes) aged 22 to 83 years were enrolled; one eye per participant was included in the analysis. If both eyes met the eligibility criteria, the eye with the higher image-quality index was selected for analysis. The inclusion criteria were age older than 18 years and the ability to provide written informed consent. The exclusion criteria were spherical equivalent outside ±3.0 diopters, best-corrected visual acuity worse than 0.8 (Snellen 20/25), media opacities, fixation instability that precluded reliable imaging, fundus disease, systemic disease likely to affect choriocapillaris perfusion (diabetes mellitus, uncontrolled arterial hypertension, coronary artery disease with a history of myocardial infarction or revascularization, previous stroke, chronic heart failure, chronic kidney disease, systemic autoimmune or inflammatory disease, hematological disorders, or malignancy), and OCTA images of insufficient quality (motion artifacts, gross segmentation errors, or projection artifacts of retinal vessels).

### 2.3. Imaging Protocol

After a standard ophthalmic examination, all participants underwent macular OCTA with a 6 × 6 mm scanning field on a DRI OCT Triton swept-source device (Topcon, Tokyo, Japan; source wavelength 1050 nm; A-scan rate 100,000 scans per second; IMAGEnet 6 software, version 1.32.18683). For each eye, a single OCTA scan was acquired; images that did not reach an image-quality index of Q ≥ 95 were reacquired, and only scans meeting this quality criterion were retained for analysis.

Segmentation of the choriocapillaris slab was performed automatically by the manufacturer’s software, with the upper boundary at Bruch’s membrane and the lower boundary 20.8 µm below Bruch’s membrane, without manual correction. This approach ensured uniform segmentation across all eyes without modification of the factory settings. OCTA images with any projection artifacts or segmentation errors were excluded from analysis.

### 2.4. Image Export and Quantitative Analysis

**Figure 1:**
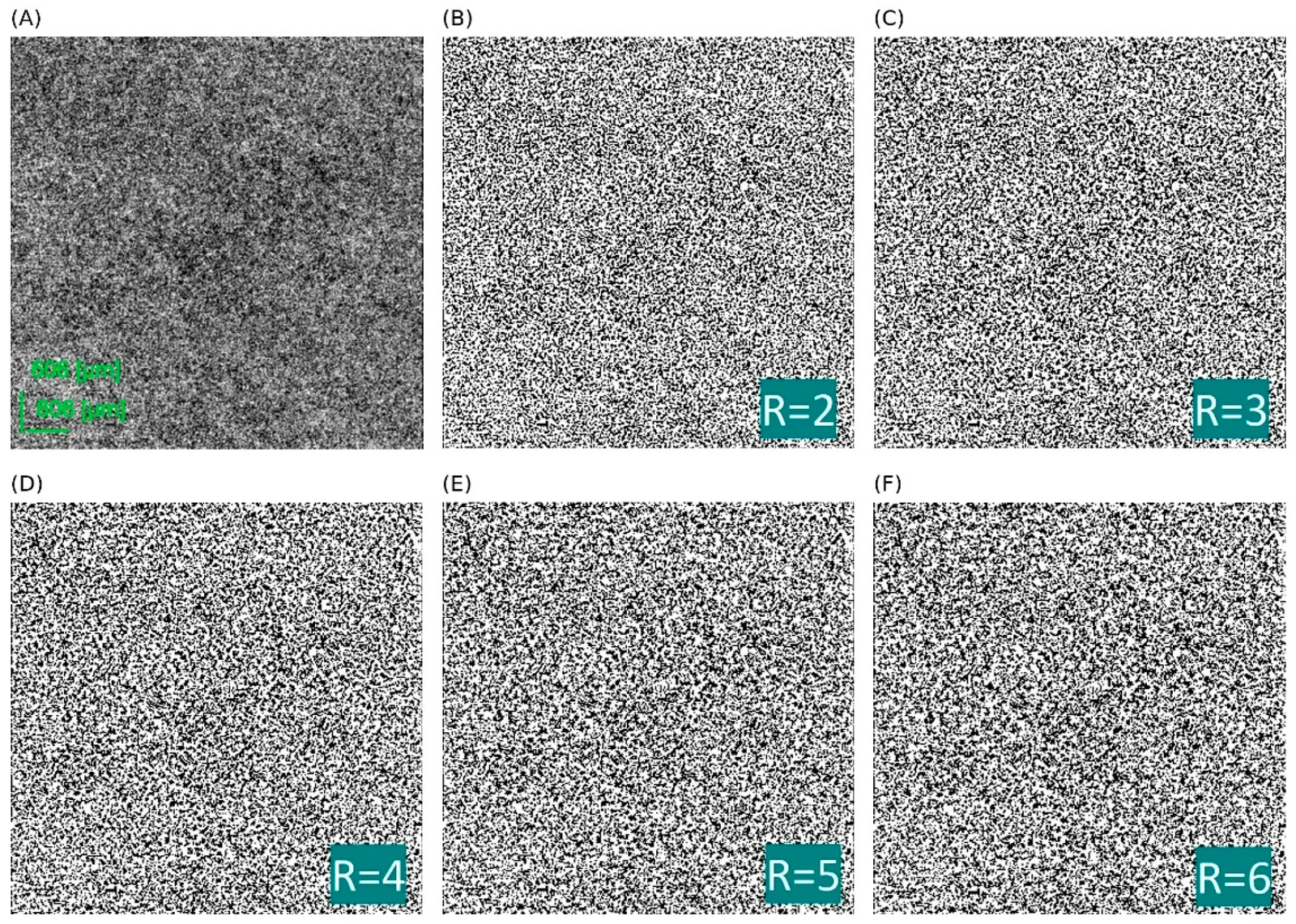
Choriocapillaris OCTA image and Phansalkar binarization at different window radii. (**A**) Representative en face optical coherence tomography angiogram of the choriocapillaris acquired with a DRI OCT Triton swept-source device (Topcon, Tokyo, Japan): 6 × 6 mm scanning field, 320 × 320 pixels, and spatial sampling of 18.75 µm per pixel. (**B**–**F**) Binarization of the image in panel A at fixed k = 0.25 and r = 0.50: (**B**) R = 2 pixels, (**C**) R = 3 pixels, (**D**) R = 4 pixels, (**E**) R = 5 pixels, and (**F**) R = 6 pixels, corresponding to radii of 37.5–112.5 µm. The fraction of white pixels representing detected OCTA flow signal decreases progressively as R increases.

Images were exported in TIFF format (320 × 320 pixels, Figure 1A) without compression and analyzed in Fiji (Fiji Is Just ImageJ; ImageJ version 1.54p, National Institutes of Health, Bethesda, MD, USA). The spatial scale was set with the Scale function according to the technical parameters of the device. The resulting spatial sampling was 18.75 µm per pixel, comparable to the average diameter of the macular choriocapillaris [1]. These images were used to quantify areas with detected OCTA flow signal and threshold-defined flow deficits, rather than anatomically confirmed nonperfusion. This sampling does not resolve individual choriocapillaris segments; resolving individual capillaries requires adaptive optics imaging [27].

Image analysis was performed in Fiji with a custom macro. The scaled choriocapillaris en face angiogram (Figure 1A) was first converted to an 8-bit image. No compensation for signal attenuation by overlying tissues (e.g., using the structural en face OCT image of the same slab) was applied, and no other preprocessing was performed before binarization. Binarization was performed with the Phansalkar local adaptive threshold. Because the Phansalkar output is highly sensitive to the window radius R, an RSWEEP mode was implemented: with k fixed at 0.25 and r at 0.50, the macro sequentially binarized the image with radii from 2 to 6 pixels (37.5 to 112.5 µm). These radii were evaluated as an empirical range of local-neighborhood scales at the present image sampling. They should not be interpreted as direct anatomical measurements or transferred to other image matrices without accounting for physical pixel size [25] (Figure 1B–F).

**Figure 2:**
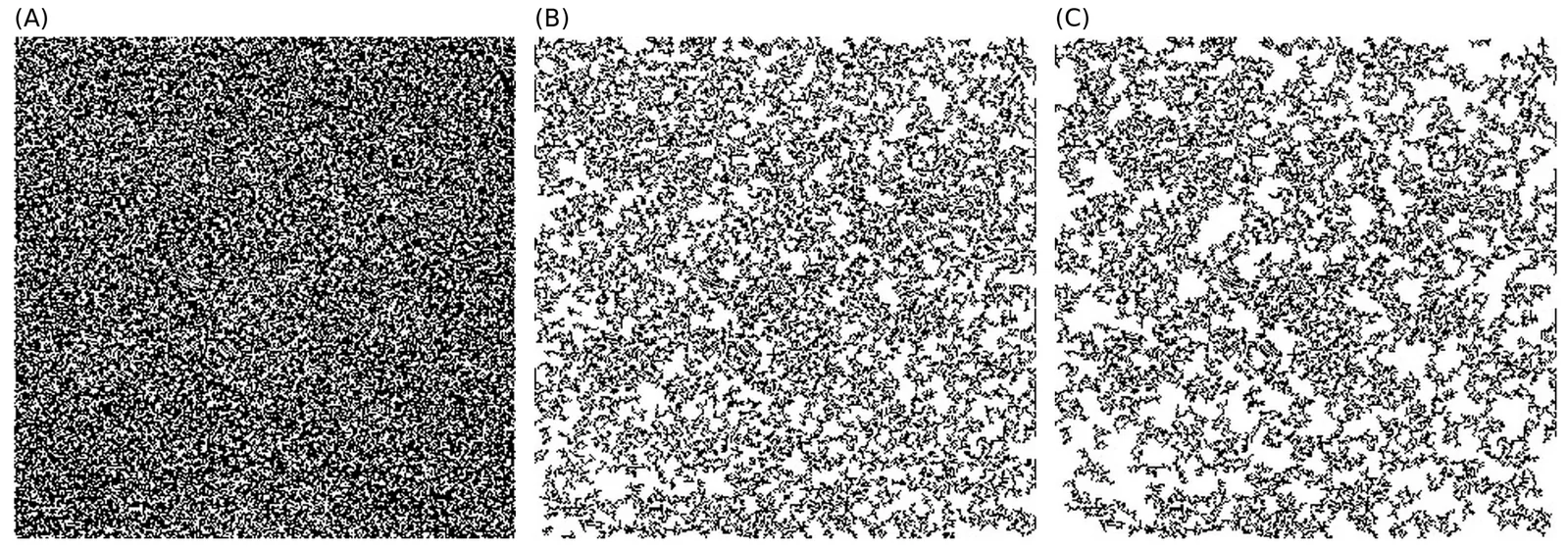
Identification and area-based filtering of choriocapillaris flow deficits. (**A**) Inverted binary mask at the illustrative RSWEEP setting of R = 3 pixels, k = 0.25, and r = 0.50; white areas represent threshold-defined flow deficits. (**B**) Flow deficits with areas exceeding 5000 µm^2^, used to derive FDN5000 and AreaFDN5000. (**C**) Flow deficits with areas exceeding 10,000 µm^2^, used to derive FDN10000 and AreaFDN10000. Retained flow-deficit regions are shown in black in panels B and C. Panels B and C were obtained by post-filtering by area the flow deficits detected with the Analyze Particles command in Fiji. No upper size limit or circularity restriction was applied.

For each radius, the full panel of biomarkers was computed: VD as the fraction of “white” pixels and FDD as the fraction of “black” pixels in the region of interest (the entire 6 × 6 mm scan field, 36 mm^2^), together with the corresponding absolute areas (vessel area and flow deficit area [FDA]). From the inverted mask (Figure 2A), flow deficits were quantified with the Analyze Particles command using a lower size limit of one pixel (≈351.6 µm^2^, corresponding to the 18.75-µm pixel size) and no upper size limit or circularity restriction. Flow deficits touching the image border were not excluded (the Exclude on Edges option was disabled). This yielded the flow deficit number (FDN, the total count of segmented flow-deficit regions) and the mean flow deficit size (MFDS, the total flow-deficit area divided by FDN). From the same particle set, the number and total area of flow deficits exceeding 5000 µm^2^ (FDN5000 and AreaFDN5000) (Figure 2B) and 10,000 µm^2^ (FDN10000 and AreaFDN10000) (Figure 2C) were derived by post-filtering individual deficits by area [28]. At 18.75 µm per pixel, these thresholds correspond to approximately 14 and 28 pixels; thus, only deficits of at least 15 and 29 pixels, respectively, were counted.

For systematic assessment of parameter effects, a krSWEEP mode was implemented. With the window radius fixed at 3 pixels (56.25 µm), two parameters were varied: k (0.10, 0.15, 0.20, 0.25, 0.30, 0.35, 0.40, 0.50) and r (0.25, 0.50, 0.75)—the normalizing coefficient of the standard deviation in the local window. This produced 24 configurations (8 values of k × 3 values of r). For each configuration, the full panel of biomarkers was recomputed.

### 2.5. Statistical Analysis

Statistical analysis was performed with IBM SPSS Statistics, version 26.0 (IBM Corp., Armonk, NY, USA) and Python 3 (NumPy, SciPy, and statsmodels). The normality of distributions was assessed within each age group with the Shapiro–Wilk test. Because some biomarkers departed from normality in the youngest group, the Kruskal–Wallis test was used as a sensitivity analysis for all omnibus comparisons. Homogeneity of variances across the three age groups was assessed using Levene’s test. Quantitative data are presented as mean ± standard deviation (M ± SD). The sex distribution across age groups was compared using the Fisher–Freeman–Halton exact test and Pearson’s χ^2^ test. Between-group comparisons across age groups were performed using one-way analysis of variance (ANOVA) with Bonferroni post hoc correction. Changes in biomarker values across window radii were summarized descriptively. Effect sizes for chronological-age group differences were quantified with η^2^ (eta-squared) for each of the 5 RSWEEP and 24 krSWEEP configurations. For the one-way ANOVA, η^2^ represents the proportion of total variance explained by age group (between-group sum of squares divided by total sum of squares). The η^2^ values were interpreted following Cohen as 0.01 (small), 0.06 (medium), and 0.14 (large). A *p* value less than 0.05 was used as the nominal significance threshold. Bonferroni adjustment refers to pairwise age-group comparisons within a configuration.

Parameter selection was exploratory and not prespecified, and no biomarker was designated a priori as the primary outcome. Because only FDN5000 and FDN10000 differed between age groups in RSWEEP, a single configuration was selected for their joint assessment using a criterion formalized after RSWEEP: the largest mean η^2^ of the two counts.

The shape of the age association of FDN5000 and FDN10000 was assessed by adding age^2^ to a linear model of centered age (F test for ΔR^2^). The mean size of large flow deficits per eye (AreaFDN/FDN) was compared between age groups using Welch’s ANOVA with Games–Howell post hoc tests. Associations of FDN10000 with FDD and AreaFDN10000 were assessed with Pearson correlation coefficients.

To account for multiple testing and for selection of the working set, *p* values from all 196 omnibus tests (28 unique configurations [5 RSWEEP and 24 krSWEEP, with the shared working set counted once] × 7 non-redundant biomarkers, with VD, vessel area, FDD, and FDA counted as one) were adjusted with the Benjamini–Hochberg false discovery rate (FDR) procedure. Selection-adjusted *p* values were obtained with a permutation max-statistic test: age-group labels were permuted 10,000 times, and the maximum η^2^ across all 196 tests in each permutation formed the null distribution. In the full cohort, parameters were selected sequentially: R in RSWEEP (k = 0.25, r = 0.50) and then k and r in krSWEEP at R = 3. Because k and r were varied only at R = 3, this sequence could not be repeated in resampled data; in each resample, the same criterion (the largest mean η^2^ of FDN5000 and FDN10000) was therefore applied in a single step across all 28 unique configurations. In the full cohort, this search selects the same working set; because it includes every configuration reachable by the sequential procedure, it is expected to be at least as prone to selection optimism. In each of 2000 bootstrap samples drawn with replacement within age groups, the configuration was re-selected; optimism was defined as its η^2^ in the bootstrap sample minus its η^2^ in the original sample, and the optimism-corrected η^2^ as the apparent η^2^ minus the mean optimism. In each of 1000 random age-stratified partitions into two halves of 35 eyes, the configuration was re-selected in one half, and the η^2^ values of FDN5000 and FDN10000 for that configuration were computed in the other (held-out) half.

## 3. Results

### 3.1. Study Cohort

A total of 70 participants (70 eyes) were enrolled and stratified into three age groups. Women accounted for 54 of the 70 participants (77.1%), and the sex distribution did not differ between age groups (Fisher–Freeman–Halton exact test, *p* = 0.729). The demographic characteristics of the cohort are presented in Table 1.

### 3.2. Window-Radius Analysis (RSWEEP)

The Shapiro–Wilk test did not detect departures from normality for FDN5000 and FDN10000 in any age group at any radius (all *p* > 0.05), whereas VD, FDD, FDA, FDN, MFDS, AreaFDN5000, and, at four of five radii, AreaFDN10000 departed from normality in the youngest group. Levene’s test did not indicate significant heterogeneity of variances across the three age groups at any radius (all *p* > 0.05). Increasing R from 2 to 6 pixels changed VD, FDD, FDA, FDN, and MFDS in the same direction in each of the 70 eyes: mean VD decreased from 62.47 ± 0.78% to 61.34 ± 0.77%, with corresponding increases in FDD (37.53 ± 0.78% to 38.66 ± 0.77%) and FDA (13.51 ± 0.28 to 13.92 ± 0.28 mm^2^); the total flow deficit number decreased from 2776 ± 229 to 2271 ± 170 (−18.2%), and MFDS increased from 4909 ± 520 to 6172 ± 588 µm^2^ (+25.7%). None of these biomarkers differed between age groups at any radius (VD, FDD, and FDA, which are algebraically linked and yield identical test statistics: *p* = 0.110–0.176, η^2^ = 0.051–0.064; FDN: *p* = 0.470–0.663, η^2^ = 0.012–0.022; MFDS: *p* = 0.316–0.504, η^2^ = 0.020–0.034). Kruskal–Wallis tests yielded the same conclusions: FDN5000 and FDN10000 differed between age groups at every radius (*p* ≤ 0.010 and *p* < 0.001, respectively), whereas the remaining biomarkers did not (*p* ≥ 0.21). Significant between-group differences were observed only for the number of flow deficits with areas greater than 5000 µm^2^ and 10,000 µm^2^ (Table 2 and Table 3).

**Table 2:** Counts and Total Areas of Large Choriocapillaris Flow Deficits by Age Group and Window Radius (M ± SD).

Biomarker(s)	Radius (R)	Group 1 (22–40 yr)	Group 2 (41–60 yr)	Group 3 (61–83 yr)	*p*
FDN5000 (*n*)	2	479.19 ± 48.95	503.32 ± 37.27	456.18 ± 52.65	0.006 *
	3	469.65 ± 41.50	496.27 ± 43.27	448.32 ± 49.63	0.003 *
	4	458.92 ± 41.07	481.14 ± 40.89	432.05 ± 52.69	0.002 *
	5	437.42 ± 40.58	459.41 ± 43.26	411.55 ± 49.46	0.003 *
	6	413.38 ± 44.75	436.68 ± 44.88	389.77 ± 46.25	0.004 *
AreaFDN5000 (mm^2^)	2	10.45 ± 0.50	10.26 ± 0.54	10.49 ± 0.68	0.364
	3	11.18 ± 0.44	11.03 ± 0.48	11.25 ± 0.61	0.381
	4	11.56 ± 0.41	11.45 ± 0.43	11.65 ± 0.55	0.359
	5	11.82 ± 0.40	11.70 ± 0.42	11.91 ± 0.54	0.310
	6	11.92 ± 0.39	11.79 ± 0.40	12.00 ± 0.52	0.275
FDN10000 (*n*)	2	244.96 ± 27.32	261.00 ± 17.47	227.82 ± 30.64	<0.001 *
	3	250.50 ± 23.00	270.09 ± 19.48	235.09 ± 28.54	<0.001 *
	4	250.62 ± 24.64	272.82 ± 20.79	237.95 ± 30.94	<0.001 *
	5	248.54 ± 24.87	270.95 ± 20.09	237.86 ± 28.55	<0.001 *
	6	242.42 ± 28.06	265.82 ± 21.30	232.05 ± 29.30	<0.001 *
AreaFDN10000 (mm^2^)	2	8.80 ± 0.62	8.54 ± 0.69	8.87 ± 0.84	0.271
	3	9.62 ± 0.59	9.43 ± 0.65	9.73 ± 0.79	0.336
	4	10.08 ± 0.54	9.96 ± 0.59	10.26 ± 0.74	0.272
	5	10.48 ± 0.52	10.34 ± 0.60	10.66 ± 0.72	0.222
	6	10.70 ± 0.52	10.56 ± 0.57	10.87 ± 0.68	0.225

FDN5000 = number of flow deficits with area greater than 5000 µm^2^; AreaFDN5000 = total area of flow deficits with area greater than 5000 µm^2^, expressed in mm^2^; FDN10000 = number of flow deficits with area greater than 10,000 µm^2^; AreaFDN10000 = total area of flow deficits with area greater than 10,000 µm^2^, expressed in mm^2^; R = window radius (in pixels). Group 1, 22–40 years; Group 2, 41–60 years; Group 3, 61–83 years. The *p* column reports the omnibus one-way ANOVA across the three age groups. These *p* values are nominal and are not adjusted across parameter configurations. Asterisks identify configurations with an omnibus *p* < 0.05 and do not identify specific significant pairs. After Benjamini–Hochberg false discovery rate correction across all 196 omnibus tests, FDN10000 remained significant at all radii (q ≤ 0.011) and FDN5000 at R = 3–5 (q = 0.049), but not at R = 2 and 6 (q = 0.063–0.064). Other results of correction for multiple comparisons are reported in Section 3.3 and Section 3.4 and Table 4. Bonferroni-adjusted pairwise comparisons for selected configurations are reported in Section 3.2 and Table 4.

**Table 3:** Omnibus One-Way ANOVA for Age-Group Differences in FDN5000 and FDN10000 at Different Window Radii.

Radius (R)	FDN5000			FDN10000		
	F	*p*	η^2^ Age	F	*p*	η^2^ Age
2	5.560	0.006 *	0.142 †	9.063	<0.001 *	0.213 †
3	6.348	0.003 *	0.159 †	11.848	<0.001 *	0.261 †
4	6.572	0.002 *	0.164 †	10.381	<0.001 *	0.237 †
5	6.420	0.003 *	0.161 †	10.286	<0.001 *	0.235 †
6	5.907	0.004 *	0.150 †	9.389	<0.001 *	0.219 †

F = F statistic; *p* = omnibus ANOVA *p* value; η^2^ age = effect size of the chronological-age factor; R = window radius in pixels; FDN5000 = number of flow deficits with area greater than 5000 µm^2^; FDN10000 = number of flow deficits with area greater than 10,000 µm^2^. The F tests have 2 between-group and 67 residual degrees of freedom. These omnibus *p* values are nominal, are not adjusted across parameter configurations (see the note to Table 2), and are distinct from the Bonferroni-adjusted pairwise comparisons. * Nominal *p* < 0.05. † Large effect size (η^2^ > 0.14).

**Table 4:** krSWEEP Configurations with the Largest Age-Group Effect for Each Biomarker and the Working Set at R = 3 Pixels.

Biomarker(s)	Configuration	k	r	η^2^	*p*	q	Significant Pairwise Comparison (*p*)
VD, FDD, FDA	Maximum η^2^	0.10	0.25	0.145	0.005	0.064	Group 2 vs. Group 3 (0.004)
	Working set	0.25	0.50	0.054	0.153	0.442	None
FDN	Maximum η^2^	0.40	0.75	0.092	0.039	0.226	None
	Working set	0.25	0.50	0.014	0.616	0.818	None
MFDS	Maximum η^2^	0.40	0.75	0.060	0.128	0.415	None
	Working set	0.25	0.50	0.022	0.469	0.715	None
FDN5000	Maximum η^2^	0.25	0.75	0.182	0.001	0.033	Group 2 vs. Group 3 (<0.001)
	Working set	0.25	0.50	0.159	0.003	0.049	Group 2 vs. Group 3 (0.002)
AreaFDN5000	Maximum η^2^	0.10	0.25	0.133	0.008	0.079	Group 2 vs. Group 3 (0.007)
	Working set	0.25	0.50	0.028	0.381	0.651	None
FDN10000	Maximum η^2^; working set	0.25	0.50	0.261	<0.001	0.008	Group 2 vs. Group 3 (<0.001); Group 1 vs. Group 2 (0.018)
AreaFDN10000	Maximum η^2^	0.10	0.25	0.119	0.015	0.119	Group 2 vs. Group 3 (0.012)
	Working set	0.25	0.50	0.032	0.336	0.621	None

VD = vessel density; FDD = flow deficit density; FDA = flow deficit area; FDN = flow deficit number; MFDS = mean flow deficit size; FDN5000 and FDN10000 = number of flow deficits with area greater than 5000 µm^2^ and 10,000 µm^2^, respectively; AreaFDN5000 and AreaFDN10000 = total area of these flow deficits; k = weighting coefficient of the local standard deviation; r = dynamic range. For each biomarker, the row “Maximum η^2^” shows the k–r combination with the largest age-group η^2^ among the 24 tested combinations, and the row “Working set” shows k = 0.25 and r = 0.50 for comparison; for FDN10000, both coincide. The working set was selected as the configuration with the largest mean η^2^ of FDN5000 and FDN10000 (Section 2.5). VD, FDD, and FDA yield identical test statistics. η^2^ describes the overall age-group effect; *p* = omnibus one-way ANOVA *p* value; q = Benjamini–Hochberg false discovery rate-adjusted *p* value across all 196 omnibus tests. Pairwise *p* values are Bonferroni-adjusted within each configuration but not across configurations; “None” indicates no pairwise *p* < 0.05. Pairwise comparisons for configurations with q > 0.05 are exploratory. Group 1, 22–40 years; Group 2, 41–60 years; Group 3, 61–83 years.

FDN5000 and FDN10000 were highest in the middle-aged and lowest in the oldest group at all radii; age-group effects were largest at R = 3 for FDN10000 (*p* < 0.001, η^2^ = 0.261) and R = 4 for FDN5000 (*p* = 0.002, η^2^ = 0.164). At R = 3, both were lower in the oldest than the middle-aged group (*p* ≤ 0.002), and FDN10000 was higher in the middle-aged than the youngest group (*p* = 0.018). R = 3 yielded the largest mean η^2^ of the two counts (0.210, compared with 0.200 at R = 4 and 0.198 at R = 5) and was therefore used for krSWEEP.

### 3.3. Parameter Analysis (krSWEEP)

In krSWEEP mode, the radius was fixed at R = 3 pixels (56.25 µm). Across the 168 krSWEEP tests (24 combinations × 7 non-redundant biomarkers), the Shapiro–Wilk test indicated a departure from normality in at least one age group in 80 tests and Levene’s test indicated heterogeneous variances in 27. Kruskal–Wallis tests confirmed 19 of the 30 nominally significant ANOVA results, including all those for FDN5000 and FDN10000; the remaining 11 (ANOVA *p* = 0.026–0.049) involved other biomarkers, and no result was significant by Kruskal–Wallis alone. The combination yielding the largest η^2^ differed between biomarkers (Table 4): k = 0.10 and r = 0.25 for VD, FDD, FDA, AreaFDN5000, and AreaFDN10000 (η^2^ = 0.119–0.145), and k = 0.25 and r = 0.75 for FDN5000 (η^2^ = 0.182). Using the same criterion as in RSWEEP, k = 0.25 and r = 0.50 yielded the largest mean η^2^ of FDN5000 and FDN10000 (0.210; next highest, 0.188) and the largest η^2^ for FDN10000 (0.261). Only the effects for FDN5000 and FDN10000 remained significant after FDR correction.

### 3.4. Shape of the Age Association and Selection-Adjusted Analyses

Adding age^2^ to a linear age model improved the fit for FDN10000 (ΔR^2^ = 0.193) and FDN5000 (ΔR^2^ = 0.171; both *p* < 0.001), whereas linear age alone was not significant (*p* ≥ 0.12); the fitted maximum was at 46 years for both counts. The mean size of large flow deficits showed the inverse pattern, being smallest in Group 2 and largest in Group 3 (deficits > 10,000 µm^2^: 35.2 ± 4.3 vs. 42.2 ± 7.6 × 10^3^ µm^2^, Welch’s ANOVA *p* = 0.001, Games–Howell *p* = 0.002; deficits > 5000 µm^2^: 22.5 ± 2.8 vs. 25.5 ± 3.9 × 10^3^ µm^2^, *p* = 0.016 and *p* = 0.015, respectively). Across eyes, FDN10000 correlated inversely with FDD (r = −0.55) and AreaFDN10000 (r = −0.46; both *p* < 0.001).

Of 38 nominally significant omnibus tests, 12 remained significant after FDR correction, all for FDN5000 or FDN10000; at the working set, q = 0.008 for FDN10000 and 0.049 for FDN5000. In the permutation max-statistic test, FDN10000 at the working set remained significant (selection-adjusted *p* = 0.001), whereas FDN5000 did not (*p* = 0.060). The working set was the configuration selected most often in bootstrap samples (32.5%); optimism-corrected η^2^ was 0.19 for FDN10000 and 0.10 for FDN5000. In split-half validation, the configurations re-selected in the training halves had a median held-out η^2^ of 0.196 for FDN10000 and 0.146 for FDN5000.

## 4. Discussion

Phansalkar local adaptive thresholding is widely used to quantify the choriocapillaris from OCTA images, but the parameters used differ substantially between studies. Previous methodological studies and a recent systematic review report that, for 6 × 6 mm scans, the window radius varies from 1 to 15 pixels across matrices of 320 × 320, 500 × 500, and 1024 × 1024 pixels, and that the values of k and r are most often either omitted or reported without justification [25,26,29]. This methodological heterogeneity limits direct comparability between studies and may hinder adoption of quantitative choriocapillaris biomarkers in clinical practice.

Previous methodological work on Phansalkar thresholding of the choriocapillaris has focused mainly on the window radius. Chu et al. varied the radius from 1 to 15 pixels on PLEX Elite images resized to 1024 × 1024 pixels while keeping k and r at the ImageJ default values, and encouraged further exploration of k [25]; Byon et al. examined how processing choices affect the repeatability of flow-deficit measurements [26]. The present study extends this work in three respects. First, k and r were varied systematically in 24 combinations rather than held at their default values. Second, the analysis was performed on native 320 × 320 exports of a widely used commercial swept-source device, at a sampling of 18.75 µm per pixel, which is coarser than in most methodological studies, and the radii were reported in physical units. Third, in addition to conventional area-based metrics, size-thresholded counts of large flow deficits were evaluated: these counts, but not VD, FDD, FDA, FDN, MFDS, or the total areas of large deficits, distinguished the age groups after correction for multiple testing, and the magnitude of this separation depended on the parameter configuration.

In the present study, we examined the influence of three key parameters of the Phansalkar local threshold—the window radius R, the weighting coefficient k, and the normalization coefficient r—on quantitative choriocapillaris biomarkers, with chronological age as the reference variable. Our findings indicate that the result of binarization, and consequently the absolute values of the derived biomarkers, depend on these parameters, consistent with previous reports [25,26]. Age was used as an internal physiological reference to compare observed associations across parameter sets, not as a ground truth for perfusion or a test of measurement repeatability or reproducibility.

The window radius defines the spatial scale of the local statistics used to compute the threshold. In our cohort, increasing R from 2 to 6 pixels (37.5 to 112.5 µm) progressively reduced FDN5000 in all age groups, whereas FDN10000 changed non-monotonically. The age-group effect sizes for both counts remained above 0.14 across the tested radius range. At R = 3, FDN10000 showed the largest observed age-group effect (η^2^ = 0.261), compared with 0.237 at R = 4. For FDN5000, the effect at R = 3 (η^2^ = 0.159) was slightly smaller than the maximum observed at R = 4 (η^2^ = 0.164). These descriptive comparisons support considering R = 3 as a practical common radius for the joint assessment of FDN5000 and FDN10000 under this protocol, without establishing that one radius is optimal for every metric. Parameter selection must also account for image sampling and thresholding behavior [25]; the physical radius of 56.25 µm should not be interpreted as a directly resolved anatomical measurement of intercapillary spacing.

The krSWEEP analysis was performed at the fixed radius of 3 pixels and therefore directly characterizes the effects of k and r at this radius. The combination k = 0.25 and r = 0.50 produced the largest observed age-group effect for FDN10000 (η^2^ = 0.261), together with an age-group effect of η^2^ = 0.159 for FDN5000. Other configurations maximized the effects for FDN5000 (k = 0.25 and r = 0.75; η^2^ = 0.182) and for VD, FDD, FDA, AreaFDN5000, and AreaFDN10000 (k = 0.10 and r = 0.25; η^2^ = 0.119–0.145); among these, only the effect for FDN5000 remained significant after FDR correction. These results demonstrate metric-dependent parameter effects. We therefore propose R = 3, k = 0.25, and r = 0.50 as a common working set for this acquisition protocol for assessing FDN5000 and FDN10000 together, rather than recommending that parameters be changed for each metric solely to maximize age-group differences.

The age pattern of the large-deficit counts was not monotonic. At every radius, FDN5000 and FDN10000 were highest in the middle-aged and lowest in the oldest group; FDN10000 was significantly higher in the middle-aged than in the youngest group at R = 3 (*p* = 0.018). Because η^2^ is insensitive to the direction and shape of group differences, the largest η^2^ values identify the configurations that best separated the age strata—chiefly the middle-aged group from the other two—rather than those most sensitive to a monotonic age-related change. The same group ordering was observed at every radius, so R, k, and r modulated the magnitude of this separation but did not create the pattern; the proposed working set is therefore a practical common configuration, not one optimized for detecting choriocapillaris aging. Ocular imaging is increasingly investigated as a source of aging biomarkers, whose clinical utility depends on their accuracy and on their associations with disease and mortality [30]. The non-monotonic pattern observed here indicates that large-deficit counts should not be interpreted as linear markers of ocular aging without longitudinal confirmation.

The lower counts in the oldest group do not indicate a smaller flow-deficit burden. Compared with the middle-aged group, the oldest group had fewer but larger flow deficits above the size thresholds, and its total area of large flow deficits (AreaFDN5000 and AreaFDN10000; Table 2) was numerically higher, although not significantly different. Across eyes, more large deficits were associated with a smaller total flow-deficit area. In a fixed scan field, size-thresholded flow-deficit counts may reflect the degree of fragmentation of threshold-defined deficit regions rather than their total area. The observed pattern, including the quadratic age association peaking at 46 years, is compatible with progressive coalescence of adjacent deficit regions, although this mechanism cannot be established from the present cross-sectional data. Selection of healthy older volunteers with high image quality and uncorrected ocular magnification may also have contributed. Counts, areas, and mean deficit size should therefore be interpreted together, and longitudinal studies are needed to test whether such coalescence occurs.

Standardized and explicitly reported binarization is also a prerequisite for the use of choriocapillaris metrics in artificial intelligence (AI)-assisted disease assessment. Retinal-imaging AI frameworks have recently been applied to multidisease detection from fundus photographs [31]. Threshold-derived OCTA features such as FDN5000 and FDN10000 could serve as interpretable inputs to, or validation targets for, such models. However, because their values depend on R, k, and r, features obtained using different binarization settings should not be pooled without appropriate harmonization or calibration.

This study has several limitations. The cohort was small and was examined at a single center with a single OCTA device, and the small number of men precluded sex-specific analyses. At 18.75 µm per pixel, individual choriocapillaris segments cannot be resolved, so the metrics describe threshold-defined flow deficits rather than capillary anatomy. At this sampling, the size thresholds of 5000 and 10,000 µm^2^ correspond to only about 14 and 28 pixels, so small differences in segmentation or binarization can split or merge adjacent deficits or move them across these thresholds; FDN5000 and FDN10000 may therefore be particularly sensitive to processing and acquisition variability. In addition, no compensation was applied for signal attenuation by the overlying retinal pigment epithelium–Bruch’s membrane complex, which is recommended when this complex is abnormal [25]. Eyes with fundus disease were excluded, but subtle age-related attenuation could still have produced artifactual low-signal regions and affected the flow-deficit metrics and their age-group differences. Axial length was not measured, and ocular magnification was not corrected. According to the Bennett formula, a 1-mm deviation from the axial length assumed by the device changes the true pixel size by about 4–5% and the pixel area by about 9% [32]. This affects all metrics expressed in absolute units, including the size thresholds and the physical window radius, and particularly the size-thresholded counts, because in a longer eye a deficit of a given true size occupies fewer pixels. The exclusion of eyes with a spherical equivalent beyond ±3.0 D limits but does not eliminate this effect, and axial-length differences between age groups could have contributed to the differences in FDN5000 and FDN10000. Most OCTA studies have not corrected the image scale [33], and axial-length-adjusted calibration has been recommended [32,34]. Systemic factors such as blood pressure and body mass index are associated with retinal vessel caliber [35]; although participants with major systemic diseases were excluded, these factors were not included as covariates, and residual confounding cannot be excluded. Parameter selection was exploratory and based on the same cohort; the permutation, bootstrap, and split-half analyses account for this only in part and do not replace independent validation. Repeated split-half validation assessed the generalizability of the data-driven selection procedure within the available cohort and should not be interpreted as independent external validation of the fixed working set. The parameters k and r were varied only at R = 3, and effect sizes were compared between configurations descriptively. Effects for biomarkers other than FDN5000 and FDN10000 did not remain significant after correction for multiple testing and should be regarded as exploratory. Test–retest repeatability was not assessed. The proposed working set is therefore a protocol-specific candidate rather than an established thresholding standard.

## 5. Conclusions

The principal finding is that R, k, and r substantially and non-uniformly affect quantitative choriocapillaris metrics. For the present acquisition protocol, the combined RSWEEP and krSWEEP findings support R = 3, k = 0.25, and r = 0.50 as a practical common working set for the joint assessment of FDN5000 and FDN10000. Chronological age served as an internal physiological reference for this exploratory, protocol-specific choice, which is neither a universally optimal nor an independently validated thresholding standard. A common, explicitly reported parameter set should be maintained for comparisons within a study instead of changing parameters solely to maximize differences for individual metrics. Validation in independent cohorts and across acquisition protocols is required before broader clinical application.

## Figures and Tables

**Table 1:** Demographic Characteristics of the Study Cohort.

Parameter	Group 1 (22–40 yr)	Group 2 (41–60 yr)	Group 3 (61–83 yr)	*p*
Participants (eyes), *n*	26	22	22	—
Age, yr (M ± SD)	30.78 ± 5.96	52.27 ± 5.71	69.55 ± 4.91	<0.001 *
Sex, male/female, *n*	6/20	6/16	4/18	0.729 †

M ± SD = mean ± standard deviation. * Statistically significant difference (*p* < 0.05, one-way ANOVA). † Fisher–Freeman–Halton exact test (Pearson’s χ^2^(2) = 0.52, *p* = 0.772).

## Data Availability

The de-identified data and image-processing macro supporting the findings of this study are available from the corresponding author upon reasonable request.

## Abbreviations

AI	artificial intelligence
ANOVA	analysis of variance
AreaFDN5000	total area of flow deficits > 5000 µm^2^
AreaFDN10000	total area of flow deficits > 10,000 µm^2^
FDA	flow deficit area
FDD	flow deficit density
FDN	flow deficit number
FDN5000	number of flow deficits > 5000 µm^2^
FDN10000	number of flow deficits > 10,000 µm^2^
FDR	false discovery rate
Fiji	Fiji Is Just ImageJ
krSWEEP	systematic variation in the Phansalkar k and r parameters
MFDS	mean flow deficit size
OCTA	optical coherence tomography angiography
RSWEEP	systematic variation in the Phansalkar window radius
SD	standard deviation
STROBE	Strengthening the Reporting of Observational Studies in Epidemiology
VD	vessel density
